# Measurements of Thermal Resistance Across Buried Interfaces with Frequency-Domain Thermoreflectance and Microscale Confinement

**DOI:** 10.1021/acsami.4c05258

**Published:** 2024-07-24

**Authors:** Ronald J. Warzoha, Adam A. Wilson, Brian F. Donovan, Andy Clark, Xuemei Cheng, Lu An, Gang Feng

**Affiliations:** † Department of Mechanical and Nuclear Engineering, United States Naval Academy, Annapolis, Maryland 21402, United States; ‡ United States Army DEVCOM Army Research Laboratory, Energy Sciences Division, Adelphi, Maryland 20783, United States; § Department of Physics, United States Naval Academy, Annapolis, Maryland 21402, United States; ⊥ Department of Physics, Bryn Mawr College, Bryn Mawr, Pennsylvania 19085, United States; ¶ Department of Mechanical Engineering, Villanova University, Villanova, Pennsylvania 19085, United States

**Keywords:** thermal boundary conductance, SiO_2_, Al_2_O_3_, sensitivity, pump−probe thermoreflectance, microscale confinement, thermal resistance

## Abstract

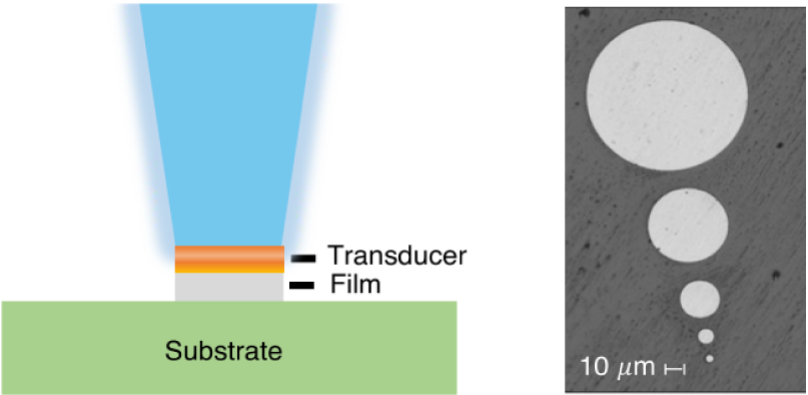

Confined geometries are used to increase measurement sensitivity to thermal boundary resistance at buried SiO_2_ interfaces with frequency-domain thermoreflectance (FDTR). We show that radial confinement of the transducer film and additional underlying material layers prevents heat from spreading and increases the thermal penetration depth of the thermal wave. Parametric analyses are performed with finite element methods and used to examine the extent to which the thermal penetration depth increases as a function of a material’s effective thermal resistance and the degree of material confinement relative to the pump beam diameter. To our surprise, results suggest that the measurement technique is not always the most sensitive to the largest thermal resistor in a multilayer material. We also find that increasing the degree to which a material is confined improves measurement sensitivity to the thermal resistance across material interfaces that are buried 10s of μm to mm below the surface. These results are used to design experimental measurements of etched, 200 nm thick SiO_2_ films deposited on Al_2_O_3_ substrates, and offer an opportunity for thermal scientists and engineers to characterize the thermal resistance across a broader range of material interfaces within electronic device architectures that have historically been difficult to access via experiment.

## 1 Introduction

Many recent studies have focused on the development of techniques to characterize the thermal resistance across interfaces that are 10s to 100s of μm below the surface of a material.^1−4^ Such interfaces are abundant in modern devices, including multilayer microelectronics packaging,^3,5^ wide bandgap materials and devices,^6,7^ power electronics architectures,^5,8,9^ and memory storage systems,^10^ and are fast becoming the largest bottleneck to sufficient heat dissipation in the next generation of these applications. Critically, interfaces at these depths are extremely challenging to characterize using existing steady-state techniques,^11−13^ which are limited to spatial resolutions above several hundred μm’s, or with advanced optical pump–probe thermoreflectance techniques, which probe at depths that range between nm’s to single-digit μm below a sample surface.^14,15^ Most recent techniques have relied on augmentations to existing thermoreflectance systems; for instance, several studies achieve larger thermal penetration depths by extending the range of modulation frequencies applied to the pump beam.^16−19^ In general, improvements in thermal penetration depth (; α is thermal diffusivity, *f* is modulation frequency) have been limited to <10 μm due to spreading in the upper transducer layer.^15^

Of particular interest in this work is the characterization of heat flow across interfaces adjacent to SiO_2_ films. SiO_2_ is fast becoming a prolific material in modern micro- and optoelectronic devices. For example, glass has been proposed as an interposer in modern 2.5D and 3D packaging architectures due to its large electrical resistivity and ease of manufacturing across large surfaces.^20−22^ Typically, metal thermal vias (e.g., copper traces) are fanned out across the interposer structure, and connected within a dielectric redistribution layer. Because the glass interposer represents a large fraction of the area across which heat can be dissipated from the active components in a chip, and materials are relatively thin, the metal-glass and glass-dielectric interfaces may be thermal bottlenecks for thermal management. Often, the thermal resistance across the glass-dielectric junction goes unused in simulations due to the large uncertainty associated with experimental measurements.^23^ Likewise, glass–glass interfaces play an important role in optoelectronic and fiberoptic systems and can act to confine heat, which elevates the temperature of the optical components and degrades their performance.^24^

Pump–probe thermal characterization techniques are capable of measuring the thermal boundary conductance across well-bonded interfaces. However, the measurement uncertainty becomes untenably large when the materials surrounding an interface are highly thermally resistive, in which case the junction is referred to as a “buried interface”. There is also ambiguity in defining a “buried interface” using a specific depth alone. The extent to which heat penetrates to some depth within a sample is largely governed by a material’s thermal resistance. Sample properties can inhibit or promote the propagation of heat into a material during a thermoreflectance measurement, and thermal anisotropy can generate an inhomogeneous temperature gradient. Consider, for example, that an equivalent temperature difference is achieved across a 1 μm thick Si layer and an ∼10 nm thick layer of SiO_2_ in the presence of a constant heat flux imposed uniformly across a boundary. A critical aspect of this work addresses deficiencies in reporting thermal penetration depths by quantifying our measurement sensitivity as a function of the ratio between the thermal resistance across the interface and the total thermal resistance of the material layers that surround it.

**Figure 1:**
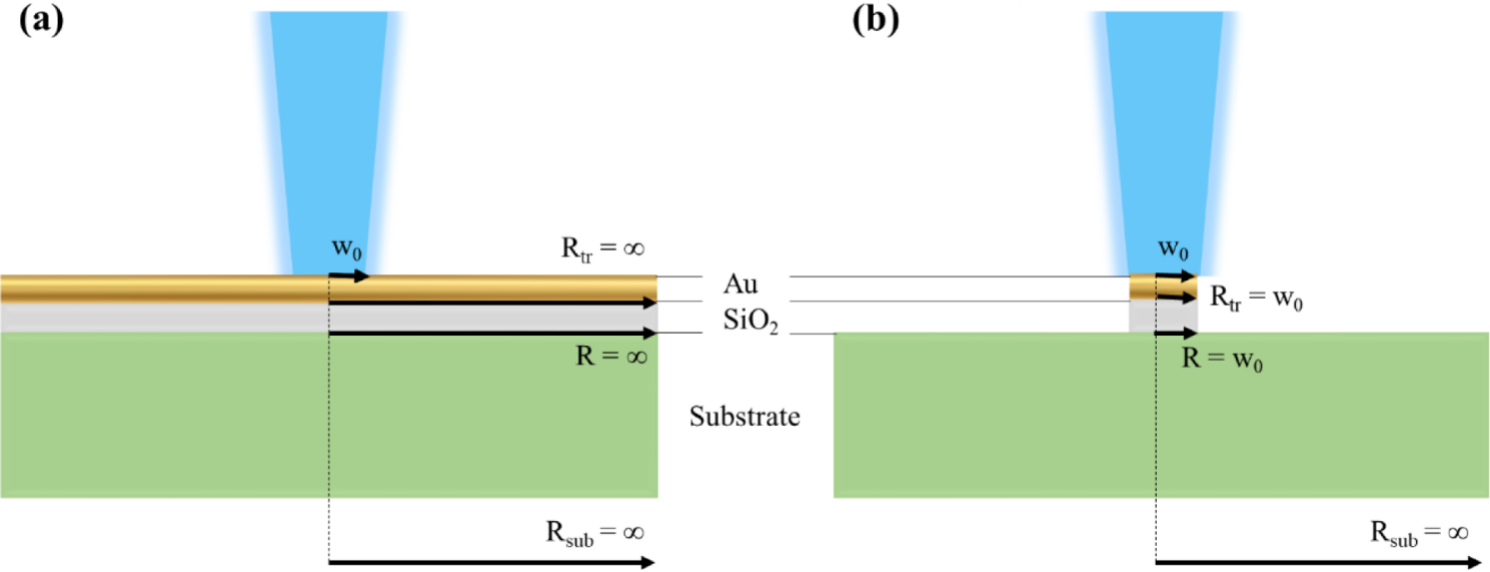
(a) Conventional semi-infinite geometry, where R/w_0_ → ∞, and (b) multilayer material whose upper layers are “confined” in a cylindrical geometry. Note that w_o_ is the pump beam radius, R is the radius of the material layer above the interface of interest, R_tr_ is the radius of the transducer layer, and R_sub_ is the radius of the substrate.

In this study we radially confine the material layers above an interface to understand how the sensitivity to interfacial thermal resistance changes as the radial dimension of the material layers above the interface approaches the diameter of our pump beam (i.e., heat source) using frequency-domain thermoreflectance (FDTR). A typical semi-infinite multilayer material is shown in Figure 1a. An accompanying schematic provides a visual representation of a material layers that are confined above an interface (Figure 1b).

This work follows a recent paper that describes a numerical fitting routine used to characterize the thermal conductivity of nonuniform geometries via FDTR.^25^ Several authors of the present study developed and validated a finite element model (FEM) in COMSOL Multiphysics to extract the thermal conductivity of a Silicon micropillar whose diameter was on the order of the pump beam. What’s more, the confinement of the pillar results in a substantial increase in the sensitivity to its thermal properties across a wide range of heating frequencies. This is a direct result of more severe temperature oscillations at the sample surface as heat reaches the pillar boundaries and is funneled into the substrate.

In this work, we conduct experiments to extract the thermal boundary conductance across an SiO_2_/Al_2_O_3_ interface and compare the magnitude and uncertainty of measured values for different pillar radii (R). These measurements provide convincing evidence of the benefits of confinement for quantifying thermal boundary conductance across “buried” interfaces with FDTR. They likewise provide a sense for the limitations of the technique.

In the following sections, we provide a brief overview of frequency-domain thermoreflectance (*Experimental Details*), details of our computational simulations and their integration into a least-squares fitting routine (*Numerical Simulations*), a description of the techniques used to synthesize SiO_2_/Al_2_O_3_ interfaces (*Materials Synthesis*), and a discussion of our parametric computational analyses and experimental observations (*Results and Discussion*).

## 2 Experimental Details

**Figure 2:**
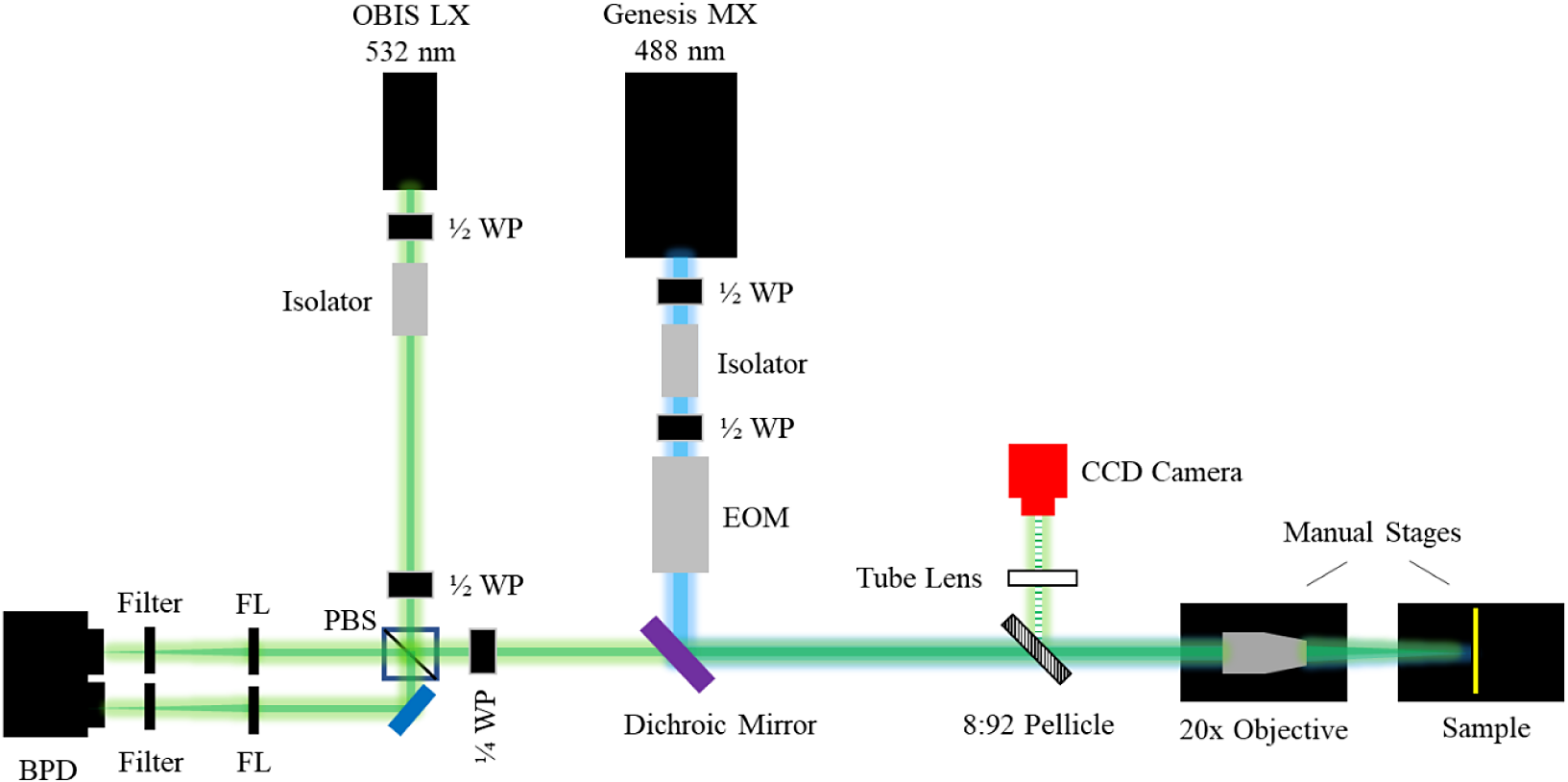
Arrangement of FDTR system in USNA Nanoscale Electronic and Thermal Transport (NEaTT) Lab. Note that EOM = Electro-optic Modulator, WP = Waveplate, BPD = Balanced Photodetector, and FL = Focusing Lens. The 8:92 pellicle beamsplitter is only inserted into the system for focusing on the sample surface with the 488 nm light blocked, which is why both the 532 nm beam and the pellicle beamsplitter are partially colored.

Frequency-domain thermoreflectance (FDTR) is the central experiment used in this study. FDTR is an optical pump–probe thermoreflectance technique that uses a pump laser to heat the surface of a sample and a separate probe laser to monitor the lag in temperature response at the sample’s surface.^26,27^ A metal transducer is usually deposited above the sample to absorb photonic energy and convert it to thermal energy, and also doubles as a thermometer given the well-known relationship between its reflectance and surface temperature.^28^ We monitor changes in reflectance at the sample surface by measuring the phase of the reflected probe beam in a photodetector with a lock-in amplifier. Very fine temperature resolution can be achieved by centering the probe wavelength at the maximum absolute value of the transducer’s coefficient of thermoreflectance (CTR). Our FDTR system uses a 488 nm pump beam and a 532 nm probe beam to optimize absorption and the CTR in an ∼80 nm Au transducer. A schematic of our FDTR technique is shown in Figure 2.

The electro-optic modulator (EOM) shown in Figure 2 is used to modulate the pump beam across a broad range of frequencies. Modulating the heating event provides access to a range of thermal penetration depths within the sample, which allows for the extraction of multiple thermal properties simultaneously in a multilayer thin-film. This technique, and the analytical framework used to extract thermal properties, is described in great detail elsewhere.^17,29^ Our signal is obtained from the reflected probe light, which is rotated 90° after twice passing through a quarter waveplate and directed into a balanced photodetector (BPD). A lock-in amplifier is used to capture the phase of the probe beam as a function of the applied modulation frequency on the pump beam. Finally, the phase of the pump beam is captured by blocking the probe beam and removing the color filter ahead of the BPD. We note that although the paper is focused on FDTR, the confinement methods we describe in the remaining parts of our paper are also expected to result in larger thermal penetration depths during time-domain thermoreflectance (TDTR) measurements. However, FDTR is capable of modulating to ultralow frequencies (<1 kHz) and can therefore achieve much larger thermal penetration depths than TDTR in practice.^15^ Thus, FDTR is more suitable for this work^i^.

## 3 Numerical Simulations

**Figure 3:**
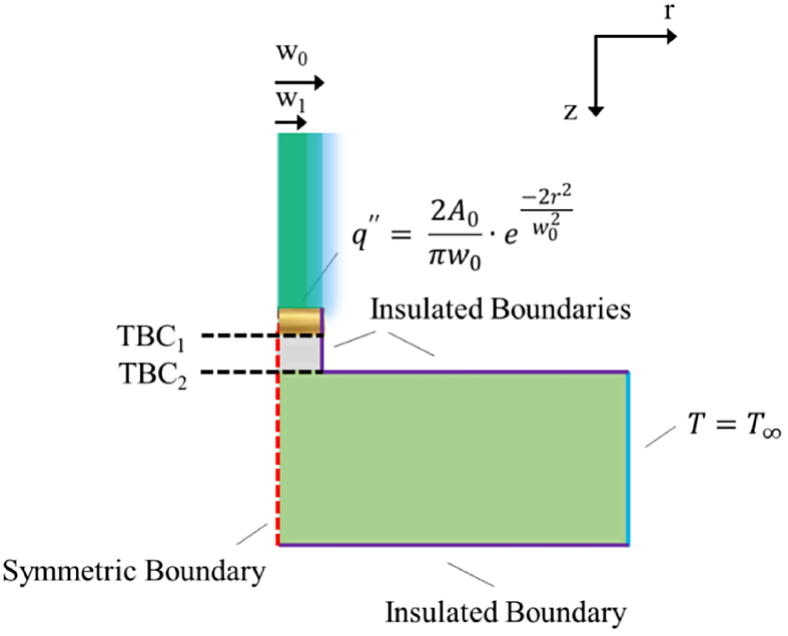
Schematic of the boundary conditions applied to the model developed in COMSOL Multiphysics (v. 5.6). Note that TBC_1_ and TBC_2_ each represent a *thermal boundary conductance*. The origin (*r* = 0, z = 0) is located at the upper left corner of the Au transducer. w_0_ and w_1_ represent the pump and probe beam radii, respectively.

We employ COMSOL Multiphysics to solve the heat diffusion equation in the frequency domain for the pillar geometries studied in this work (Figure 1b). Numerical simulations are developed in the absence of an analytical solution, which can properly account for the boundary conditions in the pillar case (unless the pillar height itself is semi-infinite.^25^ COMSOL perturbs the heat flux at the boundary to solve the heat transfer equation, represented by,
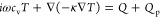
1where ω is the applied angular frequency of the pump laser, which is altered via an electro-optic modulator, *c*_*v*_ is the material’s volumetric heat capacity, T is the temperature, Q is the applied heat load, and Q_p_ is the perturbed heat load. The heat flux (q″ = Q/A) applied across the upper boundary and within the pump beam diameter is,^25^
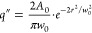
2where A_0_ is the average power of the heating beam, w_0_ is the pump beam diameter, and r is the distance from the center of the beam. The applied boundary conditions are depicted in Figure 3.

Of interest in this work is extracting the value of TBC_2_, which is the thermal boundary conductance between the film and substrate (note that thermal boundary conductance is the inverse of thermal boundary resistance, *R* = 1/TBC). In particular, we concern ourselves with measurement sensitivity to the value of TBC_2_ as the thickness (or thermal resistance) of the film layer increases.

**Figure 4:**
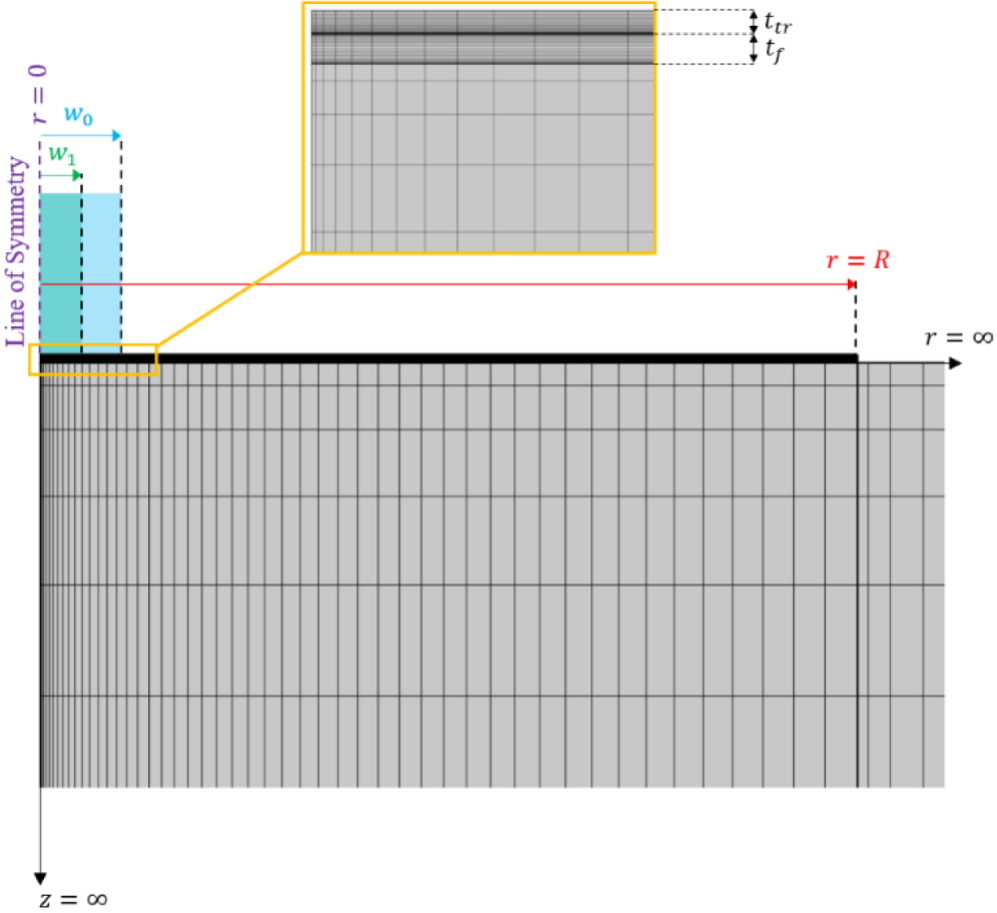
Meshed domains using graded rectangular elements. Note that *R* is the radius of the pillar, *t*_*f*_ is the film thickness, and *t*_*tr*_ is the thickness of the transducer layer. w_0_ and w_1_ remain the pump and probe radii, respectively.

A graded rectangular mesh is applied to the geometry shown in Figure 4. Here, the substrate is split into two separate domains in order to guarantee nodal alignment at the interfaces. The mesh itself is graded using a 5:1 element ratio with an arithmetic sequence such that large nodal densities are generated in close proximity to each interface. The total number of elements in each domain is fixed at 1250 (50 in the radial direction and 25 in the through-plane direction), which was determined via a mesh independence study^ii^. A depiction of the mesh used in this work is shown in Figure 4.

These numerical simulations are integrated into a least-squares fitting routine.^25^ Thermal properties of interest are defined using guess values and the error between the simulated phase lag on the sample surface and measured FDTR data is minimized. To compute the phase lag on the sample surface we use,^25,26^
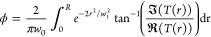
3where  and  are the imaginary and real parts, respectively, and *T* is the temperature at the sample surface. The sensitivity to a single parameter *p* can be computed as *S*_*p*_ = *∂ϕ*/*∂ln*(*p*).

We note that this technique was previously validated for a handful of semi-infinite, bilayer materials (Au/substrate) and Si micropillars having well-known thermal properties.^25^ As a result, we refer the user to this previous work for details on computational validation.

## 4 Materials Synthesis

A Veeco Fiji G2 Plasma Enhanced Atomic Layer Deposition (PEALD) system is used to deposit SiO_2_ thin films (5 nm) on Al_2_O_3_ substrates. The substrate temperature is held constant at 75 °C during deposition. Bis(diethlamido)silane (BDEAS) (CAS#: 27804-64-4, >99% from Strem Chemicals, INC.) is used as the precursor and oxygen (>99.999%) is used to generate plasma oxygen as the coreactant for our SiO_2_ films. This results in a growth rate of 0.09 nm/cycle. Au films (∼80 nm thick) are deposited using DC magnetron sputtering with a base vacuum of about 1 × 10^–8^ Torr. The 5 nm thick SiO_2_ films produced via ALD are used to gain sensitivity to the interfacial thermal resistance at the SiO_2_/Al_2_O_3_ junction. In order to demonstrate the difficulty obtaining the interfacial thermal resistance across interfaces at larger depths (and the utility of confinement), 200 nm thick SiO_2_ films are prepared via sputtering, and pillar geometries are formed using direct-write photolithography.

**Figure 5:**
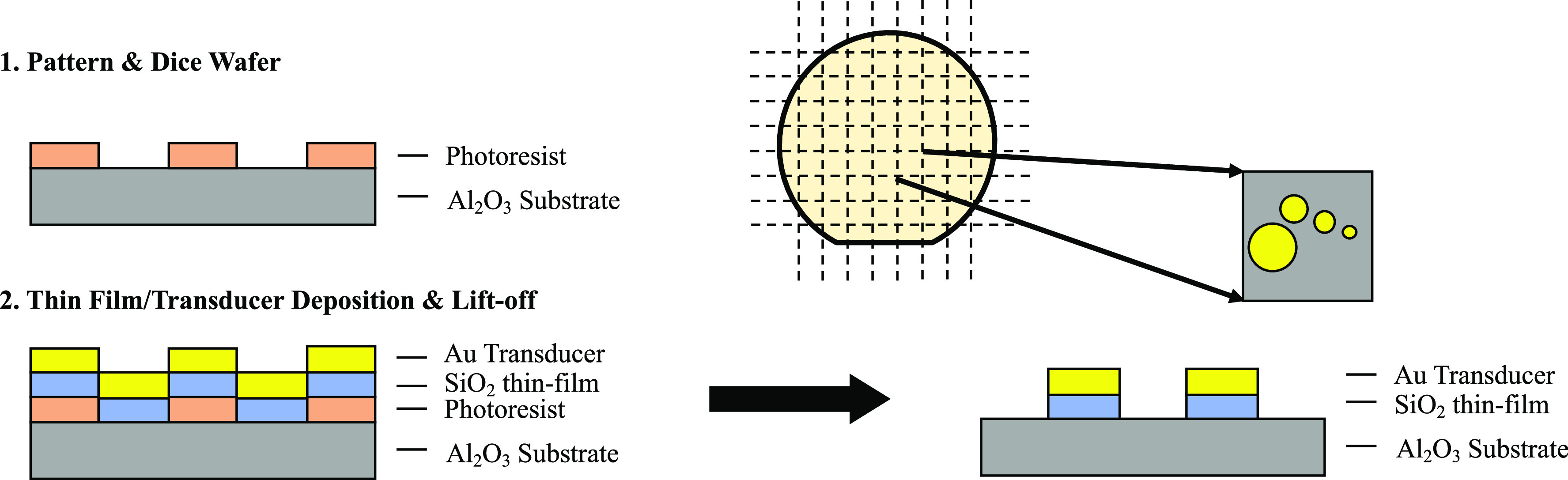
Fabrication steps for confined geometries: (1) pattern and dice the wafer, (2) deposit thin film; deposit Au transducer; liftoff. Final sample is shown on the diced wafer piece to the right, indicated by the two arrows, with the arrangement of circular pillars.

Confined geometries are produced using direct-write photolithography (Heidelberg VPG200++) with enough dose to ensure photoresist cross-linking occurs for image reversal. A flood exposure of UV and development in 1:4 diluted AZ400 K allows for the removal of photoresist where the SiO_2_ and transducer layers will be deposited. A uniform Au transducer (∼80 nm) is deposited above the sample surface using electron beam evaporation (Evatec 541 BAK system), and the sample is placed in an ultrasonic bath to remove the photoresist and lift off all unwanted metallization. This same pattern can be used with the application of positive exposure and an etching process to form pillars along the film. A 4-wave ion mill equipped with mass spectrometry is used to confine only the film in this case; the spectrometer is used to determine a rise in Si signal that corresponds to the film being fully removed. This process is shown in Figure 5. In this work, we utilize the confined film/transducer fabrication process to interrogate the TBC measurement uncertainty for the interface between the film and the substrate as a function of film (pillar) thickness. The transducer thickness is held constant at 80 nm Au/5 nm Ti.

## 5 Results and Discussion

**Figure 6:**
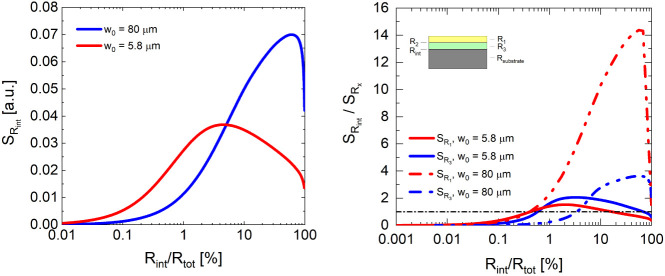
(Left) Measurement sensitivity (S_Rint_) to normalized thermal boundary resistance (TBR), where *R*_tot_ is the total thermal resistance of the multilayer material system. (Right) Normalized sensitivity (S_Rint_/S_Rx_) vs normalized TBR for 80 nm Au/2 nm Ti/1 00 nm SiO_2_/Si multilayer material system. Note that we assume the Ti layer to be part of the first interface and that R_2_ is constant and negligible relative to the other thermal resistances, which is based on details provided in ref.^25^ These sensitivities were produced using the numerical simulations described earlier.

Modern thermal characterization techniques require the presence of a thermal gradient to evaluate a material’s thermal properties. In the simplest case, a heat load is imposed at the boundary of a sample and heat is removed at the opposite end. At steady-state conditions, the thermal gradient formed across the material can then be used to extract a material’s thermal conductivity via Fourier’s Law.^12^ The presence of a thermal gradient is also required in optical pump–probe thermoreflectance measurements. A general consensus within the scientific community is that all of these measurements are most sensitive to the largest thermal resistor in a multilayer material system. However, it is unknown to what extent (and under what circumstances) this remains true for optical pump–probe thermoreflectance techniques as it has not been systematically evaluated. It is therefore useful to understand a conventional measurement’s sensitivity to the thermal resistance across a buried interface. As mentioned previously, it is difficult to quantify a depth associated with the extent to which an interface is buried due to differences in material thermal conductivity. Instead, we examine the thermal resistance of the interface as a fraction of the total thermal resistance of the material system or the thermal resistance of the layer above and adjacent to the interface, as shown in Figure 6.

Measurement sensitivities are shown for both a 5.8 μm and an 80 μm pump beam for both plots in Figure 6. In the first plot, measurement sensitivity increases as the interface’s contribution to the total thermal resistance of the multilayer material also increases. This is consistent with the intuitive claim that thermoreflectance measurements are most sensitive to the largest thermal resistor in the material system. However, there is also very clearly a threshold above which a reduction in sensitivity to the thermal resistance across an interface occurs. This indicates that if the interfacial thermal resistance is too high, the interface itself becomes insulating and forces heat to spread in the layers above it (rendering our measurement more sensitive to those upper layers, and less sensitive to the interface; see Figure S1 for additional details). This becomes quite apparent in the second plot in Figure 6, which shows the normalized sensitivity of the interface relative to an individual layer in the multilayer material system. Initially, our sensitivity to the interfacial thermal resistance increases rapidly as its contribution to the total thermal resistance also increases. However, sensitivity then decreases due in part to an increase in the sensitivity of both the transducer and the SiO_2_ layer as the interface itself becomes more resistive (and eventually “insulating”). This result itself provides critical insight for the wider thermal characterization community.

It is also instructive to consider the impact of confined geometries (i.e., *R* < ∞, where “R″ is defined in Figure 4) on measurement sensitivity to the thermal resistance across a “buried interface”. Of course, the term “buried interface” is ambiguous without some context,^15^ and largely depends on the total thermal resistance of the material system. Thus, we define a “buried interface” as one in which R_int_/*R*_tot_ ≤ 1%, where R_int_ is the thermal resistance across the interface and the total thermal resistance of the multilayer material is *R*_tot_. This definition provides an “apples-to-apples” comparison between measurement sensitivities given the propensity for spreading in the layers above the primary interface.

**Figure 7:**
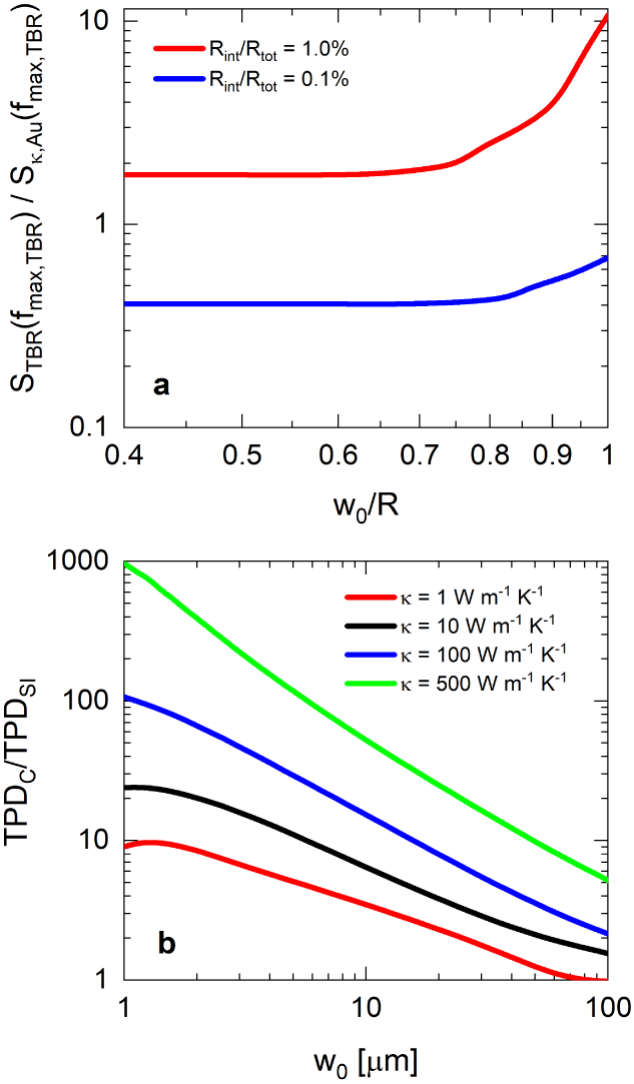
(a) TBR sensitivity relative to Au transducer layer thermal conductivity sensitivity at the frequency where ST BR is maximized as a function of transducer/thin-film confinement, and (b) 1/e thermal penetration depth of fully confined transducer/thin-film layers (TPD_C_, where *R* = w_0_) relative to 1/e thermal penetration depth of a semi-infinite multilayer system (TPD_SI_) as a function of pump beam diameter (note that all simulations for (b) were performed at *f* = 100 Hz). All sensitivities and thermal penetration depths are quantified via numerical simulations.

Figure 7a shows the impact of confinement (defined as w_0_/R) on the maximum sensitivity of our measurement to the thermal boundary resistance at a buried interface. The sensitivity to thermal boundary resistance is normalized to the sensitivity to the thermal conductivity of the transducer at the modulation frequency where the sensitivity to thermal boundary resistance is maximized. We compare these two sensitivities due to established notions that heat is likely to spread in the upper transducer layer when its thermal resistance is much less than any other layer in the material system (which causes heat to spread in the transducer layer rather than traverse the interface).^15^

The distributions plotted in Figure 7a strongly imply that confinement of the film and transducer layers increases our sensitivity to the underlying thermal boundary resistance at the film/substrate interface. However, when R_int_/*R*_tot_ ≤ 1%, the degree of confinement must be relatively high to sufficiently increase the sensitivity to R_int_ (i.e., w_0_/R > 0.7). This phenomenon is readily explained by significant enhancements in thermal penetration depth as the radial geometry becomes increasingly confined. In Figure 7b, we observe an increase in thermal penetration depth between 1 and 3 orders of magnitude as the thermal conductivity of the film layer increases and the pump diameter decreases. Note that typical pump diameters for FDTR are less than ∼30 μm;^31^ however, pump diameters approaching 100 μm are show to illustrate that heat flow can be approximated as nearly 1-D (i.e., TPD_C_/TPD_SI_**→** 1).

The magnitude of the increase in thermal penetration depth as a function of confinement is also important to consider, particularly in cases where *in situ* characterization is required.^3,32,33^ The results shown in Figure 7b suggest that this technique allows us to probe on the order of 100s of μm to several mm when *k* ≥ 100 W m^–1^ K^–1^, which is several orders of magnitude larger than the maximum theoretical TPDs predicted for semi-infinite substrates.^14,15^ In fact, these results (in tandem with results from our previous work^25^ indicate that *actual devices with unique, nonsemi-infinite geometries can be thermally characterized without modifications that may otherwise alter the underlying structure or feature distribution in any single material layer*. This is critically important for understanding the thermal behavior of future electronic devices.

**Figure 8:**
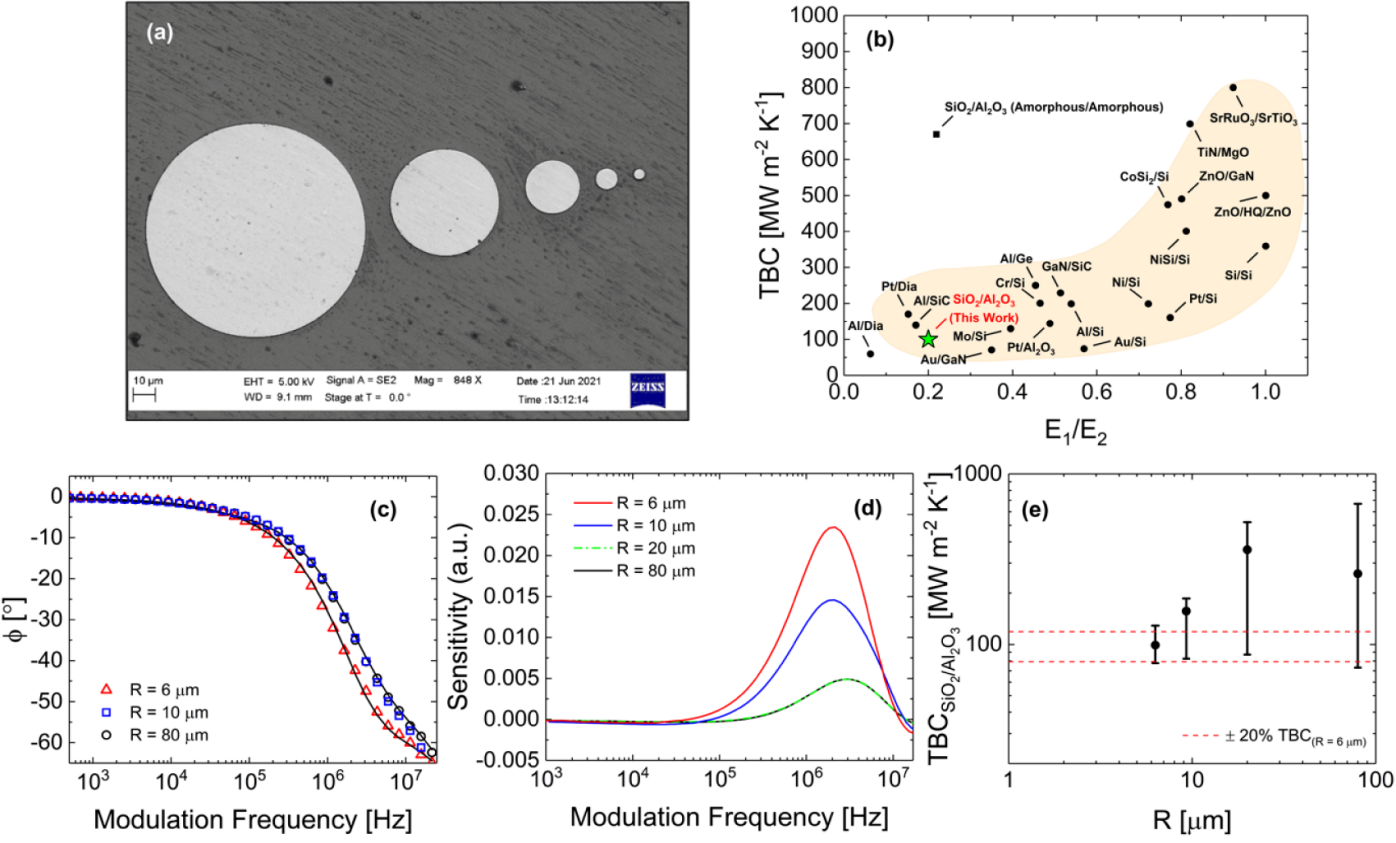
Major results showing (a) SEM image of pillars, (b) measured TBC as a function of the ratio between elastic moduli of adjacent materials (E_1_/E_2_; figure adapted from ref (34); plot shows TBC between Al/Diamond,^35^ Pt/Diamond,^35^ Al/SiC,^36^ Mo/Si,^37^ Au/GaN,^38^ Pt/Al_2_O_3_,^39^ Cr/Si,^40^ Al/Ge,^41^ Au/Si,^40^ Al/Si,^37^ GaN/SiC,^42^ Ni/Si,^37^ Pt/Si,^40^ NiSi/Si,^43^ Si/Si,^44^ CoSi_2_/Si,^43^ ZnO/GaN,^45^ ZnO/HQ/ZnO,^46^ TiN/MgO,^47^ SrRuO_3_/SrTiO_3_,^48^ SiO_2_/Al_2_O_3_ (both materials amorphous),^49^ and the measured TBC at the SiO_2_/Al_2_O_3_ (Al_2_O_3_ is crystalline) for this work, (c) modulation frequency vs phase lag for pillars with radii of 2.9 μm (red triangles), 5 μm (blue squares), and 40 μm (black circles); 10 μm pillars not shown due to overlap between the 40 and 10 μm pillar diameter data, (d) sensitivity to TBC at SiO_2_/Al_2_O_3_ interface as a function of modulation frequency for pillar radii of 2.9 μm (red line), 5 μm (blue line), 10 μm (green dashed line), and 40 μm (black line), and (e) measured TBC and uncertainties as a function of pillar diameter for SiO_2_/Al_2_O_3_ interfaces fabricated in this study. Note that pillar radii are measured via SEM as in ref (25), with an uncertainty of ±0.1 μm. Pump and probe radii for all measurements are fixed at 2.85 and 2.65 μm, respectively.

Finally, we characterize the TBC at the SiO_2_/Al_2_O_3_ interface for a confined, 200 nm thick SiO_2_ film and compare it with measurements across a 5 nm thick film. In this work, a 2.85 μm pump radius is used to measure TBC for pillars with radii of 2.9 μm, 5 μm, 10 and 40 μm, as shown in the scanning electron microscopy (SEM) image in Figure 8a. Pillar diameters are measured via SEM and heights are confirmed with profilometry. The 200 nm thick film is used to achieve a ratio of R_int_/*R*_tot_ ≤ 1.0%. We expect the TBC at the SiO_2_/Al_2_O_3_ interface to be between 100 and 300 MW m^–2^ K^–1^ based on measurements of thermal boundary conductance as a function of the ratio between each material’s elastic moduli (= 0.2, Figure 8b), and a measured TBC of 121 MW m^–2^ K^–1^ across the 5 nm film. The measured phase lag and corresponding sensitivities are plotted in Figure 8c,d for each of the aforementioned pillar radii, and the results for measured TBCs are provided in Figure 8e.

The measured data are shown in Figure 8c and provide clear evidence of a deviation in phase lag once the pillar is fully confined. This confinement forces the heat to traverse the interface where it otherwise would not, which results in an increased sensitivity to the measured value of the TBC (99.8 MW m^–2^ K^–1^), as shown in Figure 8d. Notably, the sensitivity to the measured TBC no longer changes as the pillar radius becomes much larger than the pump diameter. Critically, the uncertainty of the measurement increases significantly as the pillar diameter increases (from ∼22% to ∼200%), principally due to the difficulty associated with forcing heat to cross the interface when the thermal resistance of the upper layer is large relative to the thermal resistance of the interface. Thus, without any geometric confinement, it would not be possible to measure the thermal boundary conductance (or interfacial thermal resistance) across a “buried” interface with any reasonable degree of certainty. This is extremely critical for measurements of heat flow across interfaces in multilayer devices whose interfaces lie well below the surface. All uncertainties are determined using deviations in transducer thickness (±5 nm), pillar height (±5 nm), pillar radius (±0.1 μm), pump and probe radii (±0.1 μm), and the top-side thermal boundary conductance (±13 MW m^–2^ K^–1^ for a measured TBC of 113 MW m^–2^ K^–1^ for the same transducer on an SiO_2_ substrate.^25^ We therefore show that geometric confinement can be used to measure the thermal boundary conductance across a buried interface due to an enhancement in measurement sensitivity. This technique can be utilized for measurements of *as-fabricated* interfaces, without the need to scale down and/or apply different processing techniques in order to decrease film thickness (each of which can alter the TBC at the interface).

## 6 Conclusion

This work shows that geometric confinement allows for a drastic improvement in the measured thermal boundary conductance across a buried interface, which we define for the first time as a percentage of the overall thermal resistance of the multilayer material system (in this study, we require R_int_/*R*_tot_ ≤ 1% to be considered a buried interface). We use a patterning and ion bombardment technique to create 200 nm thick SiO_2_ pillars on an Al_2_O_3_ substrate with varying pillar radii, and characterize the thermal boundary conductance at the interface using frequency-domain thermoreflectance (FDTR). A recently developed numerical technique is applied to account for the finite boundaries of the pillars relative to the pump radius. Results show that geometric confinement forces the heat from the pump beam to travel much further into the sample, which results in a much lower uncertainty for measurements of TBC across buried interfaces. These results are critical for uncovering the mechanisms that govern heat flow across relevant electronics packaging interfaces.

## Supporting Information Available

Calculation of thermal resistances and shape factor and thermal resistance for each material layer and interface in the experimental material system (PDF)

## Footnotes

i We note that steady-state thermoreflectance^2^ might achieve thermal penetration depths on par with those encountered in low-frequency FDTR measurements. We choose to avoid redundancy in this work by focusing exclusively on FDTR.

ii Our mesh independence study examined the phase lag distribution generated at the top of the sample surface for a semi-infinite, 80 nm Au transducer/fused silica substrate characterized in ref.^30^ The phase lag at all frequencies was required to match to within 1% of measured values.
