# Supplementary material for: Measurements of Thermal Resistance Across Buried Interfaces with Frequency-Domain Thermoreflectance and Microscale Confinement
Source: ACS Appl Mater Interfaces. 2024 Jul 24;16(31):41633–41. doi: 10.1021/acsami.4c05258 (PMC11310922; doi:10.1021/acsami.4c05258)
Supplement: Supplementary file 1 — am4c05258_si_001.pdf [file am4c05258_si_001.pdf]

Supporting Information: Measurements of Thermal Resistance Across Buried Interfaces with Frequency-domain Thermoreflectance and Microscale Confinement

Ronald J. Warzoha[1], Adam A. Wilson[2], Brian F. Donovan[3], Andy Clark[4], Xuemei Cheng[4], Lu An[5], and Gang Feng[5]

[1]Department of Mechanical and Nuclear Engineering, United States Naval Academy, Annapolis, MD, 21402

[2] US Army DEVCOM Army Research Laboratory, Energy Sciences Division, Adelphi, MD 20783.

[3] Department of Physics, United States Naval Academy, Annapolis, MD, 21402

[4] Department of Physics, Bryn Mawr College, Bryn Mawr, PA, 19085

[5] Department of Mechanical Engineering, Villanova University, Villanova, PA, 19085

---

[1] Author to whom correspondence should be addressed.

Calculation of Thermal Resistances

Because heat spreads radially and traverses the through-thickness direction of the films and substrate, and because heat is applied non-uniformly at the top of the film, we find it necessary to numerically compute the total thermal resistance of the film stack to project an expected ratio of Rint/Rtot.

To do this, we calculate the shape factor (*S*) for each layer in the material stack using Eqn. S1:

$$S = \frac{q}{k\Delta T} \quad \text{(S1)}$$

where ΔT is the difference between the average temperature at the upper boundary and the average temperature at the lower boundary of an individual layer, q is the applied heat load, and k is the thermal conductivity of the material layer. The shape factor can be used to express a 2-D thermal resistor via Eqn. S2:

$$R = \frac{1}{Sk} \quad \text{(S2)}$$

Simulations can be run at any frequency to compute individual thermal resistances and obtain a total thermal resistance (which includes the thermal resistance at each interface). An 80 nm Au/200nm SiO_2/Al_2O_3 multilayer material is built in COMSOL Multiphysics. In this case, the pump and probe radii are 5.85 μm and 2.85 μm, respectively. The thermal boundary resistance between the Au and SiO_2 is assumed to be on the order of 200 MW/m^2K (consistent with the use of a nm thick Ti adhesion layer[2]), and the absorbed power at the surface is 25 mW. To compute each value of *S* at a steady-state, we apply a DC heating event at the sample surface (i.e., $f = 0$ Hz). In the measurement, the contribution made to the total thermal resistance by the substrate will decrease as the heating frequency increases. This is principally due to the fact that the thermal penetration depth is constrained when heat is modulated. However, we desire to remain consistent with fundamental heat transfer physics and simulate at a steady state. The computed value of *S* for each material layer and the associated thermal resistance is provided in Table S1.

Table S1. Shape factor and thermal resistance for each material layer and interface in the experimental material system.

Material Layer	Shape Factor, *S* [m]	Thermal Resistance, *R* [m^2K/W]
80 nm Au	0.540	$2.80\cdot10^{-9}$
Au/SiO_2 Interface	-	$5.00\cdot10^{-9}$
200 nm SiO_2	1.569	$1.62\cdot10^{-7}$
SiO_2/Al_2O_3 Interface	-	$8.26\cdot10^{-9}$
Al_2O_3 Substrate	0.006	$1.41\cdot10^{-6}$

Thus, the thermal resistance of the SiO_2/Al_2O_3 interface represents R_{int}/R_{tot} = 0.52%. This information has been added to a separate Supporting Information document.

[2] Olson, D.H., Freedy, K.M., McDonnell, S.J. and Hopkins, P.E., 2018. The influence of titanium adhesion layer oxygen stoichiometry on thermal boundary conductance at gold contacts. *Applied Physics Letters*, *112*(17).

For the above configuration, we also plot the sensitivity to the Au film's thermal resistance, the SiO_2/Al_2O_3 interfacial thermal resistance, and the SiO_2 film's thermal resistance in support of our assertion that the sensitivity to the Au and SiO_2 thermal resistance increases as the SiO_2/Al_2O_3 interface becomes more insulating. The expected contribution of the SiO_2/Al_2O_3 interface to the total thermal resistance is also plotted as a vertical line to highlight the low measurement sensitivity to G_2 for a blanket, semi-infinite film.

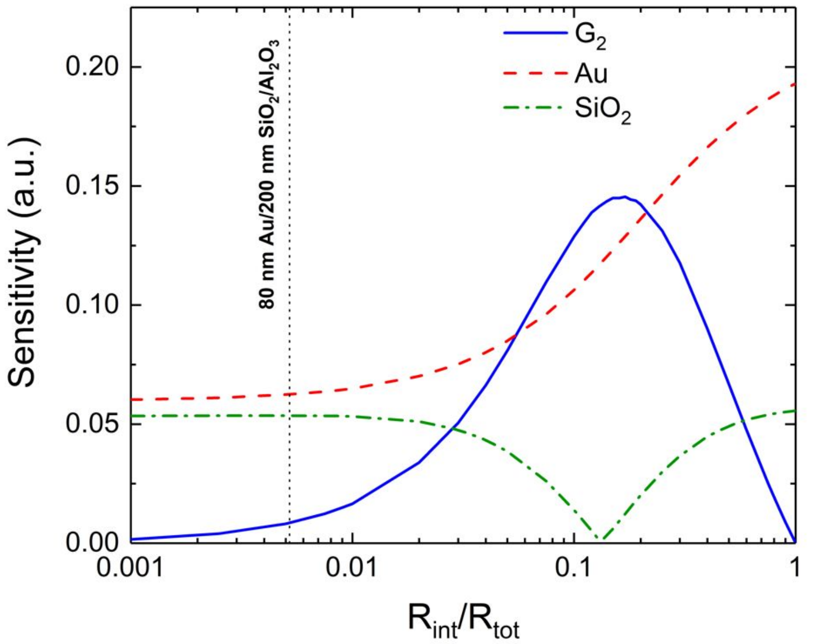

Fig. S1: Fraction of thermal resistance as a function of total thermal resistance vs. sensitivity for an 80 nm Au/200 nm SiO_2/Al_2O_3 film
